# The 2.0 Å X-ray structure for yeast acetohydroxyacid synthase provides new insights into its cofactor and quaternary structure requirements

**DOI:** 10.1371/journal.pone.0171443

**Published:** 2017-02-08

**Authors:** Thierry Lonhienne, Mario D. Garcia, James A. Fraser, Craig M. Williams, Luke W. Guddat

**Affiliations:** The School of Chemistry and Molecular Biosciences, The University of Queensland, Brisbane, QLD, Australia; Griffith University, AUSTRALIA

## Abstract

Acetohydroxyacid synthase (AHAS) catalyzes the first step of branched-chain amino acid biosynthesis, a pathway essential to the life-cycle of plants and micro-organisms. The catalytic subunit has thiamin diphosphate (ThDP) and flavin adenine dinucleotide (FAD) as indispensable co-factors. A new, high resolution, 2.0 Å crystal structure of *Saccharomyces cerevisiae* AHAS reveals that the dimer is asymmetric, with the catalytic centres having distinct structures where FAD is trapped in two different conformations indicative of different redox states. Two molecules of oxygen (O_2_) are bound on the surface of each active site and a tunnel in the polypeptide appears to passage O_2_ to the active site independently of the substrate. Thus, O_2_ appears to play a novel “co-factor” role in this enzyme. We discuss the functional implications of these features of the enzyme that have not previously been described.

## Introduction

**Fig 1:**
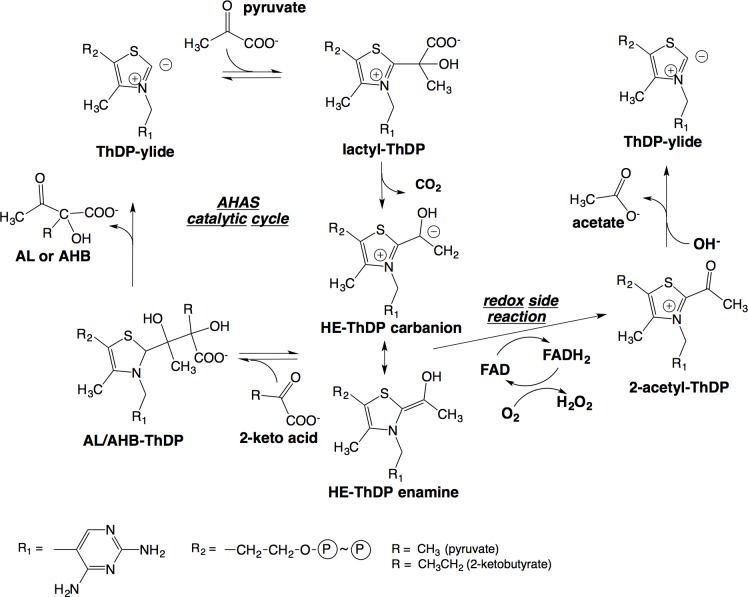
Catalytic cycle and redox side-reaction of AHAS with key intermediates. ThDP = thiamin diphosphate, HE = hydroxyethyl, AL = 2-acetolactate, AHB = 2-acetohydroxybutyrate.

Acetohydroxyacid synthases (AHAS) catalyses the first step in *de novo* branched-chain amino acid (BCAA) biosynthesis, a pathway present in plants, fungi, algae and bacteria. It is the target for more than 50 commercial herbicides that are a foundation in weed control for all major crops. AHAS catalyses the condensation of pyruvate with another molecule of pyruvate, or with 2-ketobutyrate, to produce 2-acetolactate or 2-acetohydroxybutyrate, respectively (Fig 1). Thiamin diphosphate (ThDP), flavin adenine dinucleotide (FAD), and a divalent metal ion are all essential co-factors in this reaction. ThDP plays a central role by forming covalent bonds with the reaction intermediates [1] (Fig 1). FAD is also absolutely required by AHAS for catalysis, although its involvement in a redox reaction has not been established [1–4]. Tittmann and co-workers^4^ have shown that in a side-reaction of AHAS, the catalytic intermediate 2-(hydroxyethyl)-ThDP (HE-ThDP) in the carbanion/enamine form can transfer two electrons to the adjacent FAD in an intramolecular redox reaction yielding 2-acetyl-ThDP and reduced FAD (Fig 1), a redox reaction similar to that for pyruvate oxidase (POX) [4]. This led to the assumption that AHAS and POX share a common POX-like ancestor that catalyses a reaction that relies on electron transfer between ThDP and FAD [4]. However, as this reaction is apparently non productive in AHAS, the current view has been that the FAD in AHAS is a vestigial remnant from a POX/AHAS ancestor with the ascribed role to maintain structural integrity.

AHAS also possesses an oxygenase side-reaction [5] that results in the ligation of a molecule of oxygen to the HE-ThDP intermediate to produce peracetate. This reaction represents ~1% of the AHAS activity^4^ and based on a competitive enzymatic assay with O_2_ and pyruvate, it has been concluded that O_2_ uses a path to access the active site that is different from the one used by the acceptor substrate [5].

Herein, an extensive high resolution X-ray analysis of the structure of *Saccharomyces cerivisiae* AHAS (*Sc*AHAS) provides new data that allows us to track the path of the O_2_ molecules as they enter a tunnel leading towards the active site. O_2_ is trapped by the enzyme in a binding site that is nearby to ThDP and has a structure that is completely analogous to a putative O_2_ binding site in glyoxylate carboligase. We also show that FAD takes different conformations in each of the catalytic centre of the dimer, suggesting the possibility that it is involved in a redox reaction.

## Materials and methods

### Protein preparation

The expression and purification of *Sc*AHAS was carried out in two steps as described previously (IMAC and gel-filtration) [6] however with a modification: the gel-filtration purification step was performed on a Sephacryl S200 size-exclusion FPLC column and *Sc*AHAS was eluted in buffer that contained 0.2 M potassium phosphate pH 7.2, 1 mM DTT and 10 μM FAD. Using a Millipore centricon with a 30 kD cut-off, *Sc*AHAS was then concentrated to 35–64 mg/ml and aliquots were snap cooled at –80°C. This process led to highly concentrated enzyme (480–870 μM) carrying the cofactor FAD, and giving an enzyme solution in which the ratio of enzyme-bound FAD *vs* free FAD is ≥ 40. Enzyme concentrations were determined by analysis using a Direct Detect spectrophotometer (Millipore).

### Crystallization and structure determination

Crystallization *Sc*AHAS was prepared as described previously [6]. Crystals were obtained by the hanging drop vapour diffusion method. The drops consisted of equal volumes (1 μl) of well solution and enzyme. For the *Sc*AHAS-pyruvate complex, pyruvate powder was added to drops containing crystals of free *Sc*AHAS and incubation was allowed for 2 hours or 3 days before crystals were cryo-cooled. Prior to cryo-cooling the crystal was transferred to a drop containing well solution, 25% glycerol, and 25% MPD.

Data were collected at the Australian synchrotron, beam line MX1 (wavelength 0.9537 Å) using the remote access program Blu-Ice [7]. The data were integrated using the program XDS [8] and scaled and merged using Aimless [9]. The phasing was determined by molecular replacement using PHASER [10] and the protein coordinates for *Sc*AHAS [11] (PDB code 1JSC). Refinement and model building were achieved using Phenix 1.8–1.9 [12] and COOT 0.7 [13], respectively. Coordinates and structure factors have been deposited in the protein data bank with accession codes of 5IMS. The O_2_ tunnel in CC_1 was analysed using MOLEonline 2.0 (http://mole.upol.cz). All structural images were generated with CCP4mg [14], except for the images showing the backbone movement of AHAS subunits and the solvent access channel generated by MOLEonline 2.0 which were generated with the PyMOL Molecular Graphics System (2002, http://www.pymol.org), DeLano Scientific, Palo Alto, CA, USA.

## Results and discussion

The structure of *Sc*AHAS was determined to 2.0 Å resolution (Table 1), a significant improvement compared to the previously published structure (2.65 Å) [11]. Crucially, this new data allows for the first time a detailed visualization of all of the active site components.

**Table 1:** Data collection and refinement statistics for *Sc*AHAS.

	*Sc*AHAS
**Crystal parameters**	
Unit cell *a*, *b*, *c* (Å)	95.68, 110.18, 180.01
Space group	*P*2_1_2_1_2_1_
Diffraction data*	
Temperature (K)	100
Resolution range (Å)	48–2
Observations	706465 (32632)
Unique reflections	130638 (6212)
Completeness (%)	99.3 (96)
R_merge_	0.075 (0.645)
R_pim_	0.034 (0.299)
〈I〉/〈σ(I)〉	11.0 (2.1)
**Refinement statistics**	
Resolution limits (Å)	48–2
*R*_*work*_	0.1656
*R*_*work*_ (highest resolution)	0.2379
*R*_*free*_	0.1925
*R*_*free*_ (highest resolution)	0.2609
rmsd bond length (Å)	0.016
rmsd bond angle (°)	1.485
Clashscore^d^	5.68
**Ramachandran plot (%)**	
Favoured	97.6
Outliers	0.5
**Contents of asymmetric unit**	
Protein chains	2
Acetate	3
O_2_	9
Mg^2+^	2
FAD	2
ThDP	2
K^+^	2
PO_4_^3-^	1
Water molecules	813
**B-factors** (Å^2^)	
Overall structure	44.53
polypeptide	44.3
Chain A	34.76
Chain B	53.17
Loop 1 (chain A, P114–P119)	26.87
Loop 1 (chain B, P114–P119)	30.47
Loop 2 (chain A, V161-P165)	21.59
Loop 2 (chain B, V161-P165)	22.86
FAD (chain A)	36.76
FAD (chain B)	45.01
ThDP (chain A)	23.33
ThDP (chain B)	31.46
Acetate (chain A)	54.36
Acetate (chain B)	60.76
Acetate (chain B)	65.22
O_2_(I) (chain A)	70.58
O_2_(II) (chain A)	58.2
O_2_(I) (chain B)	85.2
O_2_(II) (chain B)	68.92
Average water molecules	47.67

* Values in parenthesis are for the outer-resolution shells (2.01–1.98 for *Sc*AHAS)

*R*_*merge*_ = ∑_*hkl*_∑_*i*_|*I*_*i*_(*hkl*) − (*I*(*hkl*))|/∑_*hkl*_∑_*i*_*I*_*i*_(*hkl*)

$R_{p.i.m.}=\sum_{hkl}\;{\left[\frac{1}{\left[N(hkl)-1\right]}\right]}^{1/2}\sum_{i}|I_{i}(hkl)-(I(hkl))|/\sum_{hkl}\sum_{i}I_{i}(hkl)$

where *I*_*i*_(*hkl*) is the observed intensity and is the average intensity obtained from multiple observations of symmetry related reflections.

^*d*^ Clashscore is defined as the number of bad overlaps ≥ 0.4 Å per thousand atoms.

### Homodimer asymmetry

**Fig 2:**
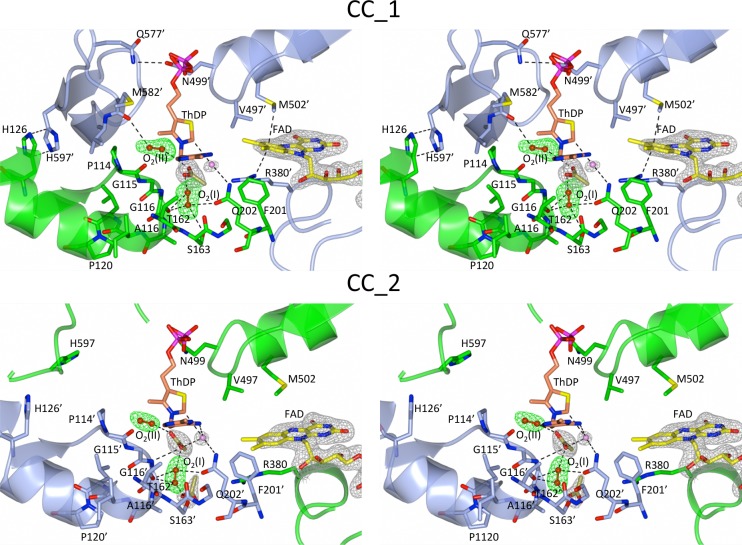
Stereo views of the structure of the *Sc*AHAS homodimer showing that different conformations occur in the catalytic centres, CC_1 and CC_2. The two polypeptide chains are coloured green and ice blue. The pink sphere represents a water molecule. Dashed black lines represent interactions occurring between the different molecules in the active site, including hydrogen bonds, van der Waals and hydrophobic interactions. Green electron densities (contoured at 3.5 σ in the F_o_—F_c_ map) correspond to the two O_2_ molecules. Grey electron densities (2F_o_—F_c_ map) correspond to acetate and FAD molecules contoured at 1.5 σ.

**Fig 3:**
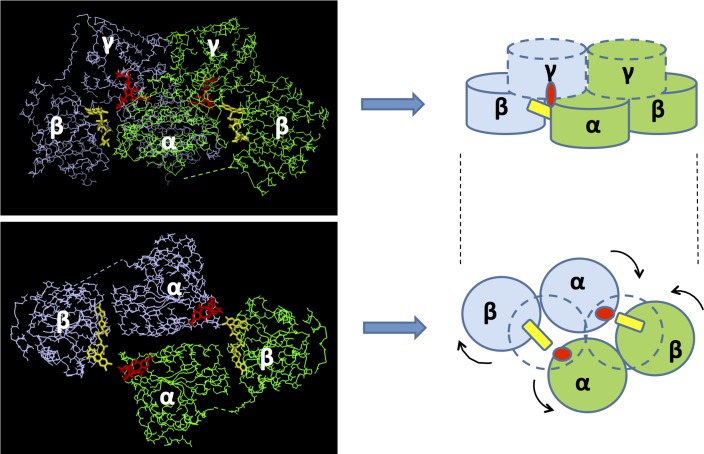
The structure of the *Sc*AHAS homodimer is asymmetric. Left images represent the polypeptide of *Sc*AHAS showing the configuration of the α, β, and γ-domains of the two subunits (green and blue). In the lower panel, the γ-domains have been removed from the image in order to allow visualization of the asymmetry of α and β-domains in the two subunits. Cartoons on the right schematize the configuration of the domains. Yellow rectangles represent FAD, and red ellipses represent ThDP.

The most obvious feature is that the two catalytic centres of the homodimer have distinct conformations (Fig 2). In the catalytic centre defined as CC_1, the isoalloxazine ring of FAD is bent by 21° across the N5-N10 axis, a conformation that increases its redox potential and thus stabilizes its reduced form [FAD_red_, either FADH_2_ or FADH¯)] [15]. In the alternate catalytic centre, defined as CC_2, the isoalloxazine ring is near to planar, a conformation expected to represent oxidized FAD (FAD_ox_) or semi-reduced FAD (FAD_radical_, either FAD¯ or FADH**˙**) [16]. Another major difference is an ordered region of polypeptide (residues 577 to 597) only visible in CC_1 that is stabilized by interactions involving H597 and H126, and Q577 and D499. These features are reflected by the general asymmetry observed in the dimer (Fig 3) suggesting that the enzyme operates similar to that a two-stroke engine with the two active sites performing identical cycles but being out of synchronization with respect of each other. Because both active sites undergo an identical catalytic cycle, it can be suggested that the two conformations observed in the crystal structure represent two different stages of the catalytic cycle at a single time point. The intrinsic nature of the crystal implies that the asymmetric configuration of the complex is uniform, suggesting that catalysis is achieved through allosteric communication between subunits. Coordination between the two active sites is consistent with the nature of the AHAS reaction that involves two stages: (i) decarboxylation of the first incoming substrate (pyruvate) to form a catalytic intermediate attached to ThDP (HE-ThDP, Fig 1), and (ii) the ligation of a second incoming substrate (pyruvate or 2-ketobutyrate) to HE-ThDP giving product.

Importantly, the structure suggests that two different redox states of FAD occur in the dimer, suggesting that it is involved in a redox reaction.

### Molecular oxygen (O_2_) is a cofactor

In each catalytic center of this structure we could fit a molecule of acetate (two in CC_2), a component of the crystallization buffer [6], and two molecules of O_2_ (O_2_(I) and O_2_(II)) to the electron density near to the reactive C2 carbon of ThDP (Fig 2). One acetate molecule fits between the O_2_ molecules, having a credible role in stabilizing the catalytic centres. It could be argued that two disordered water molecules could be modeled into the density occupied by the O_2_ molecules, however, strong support for the assignment of O_2_ to these sites is justified as follows.

**Fig 4:**
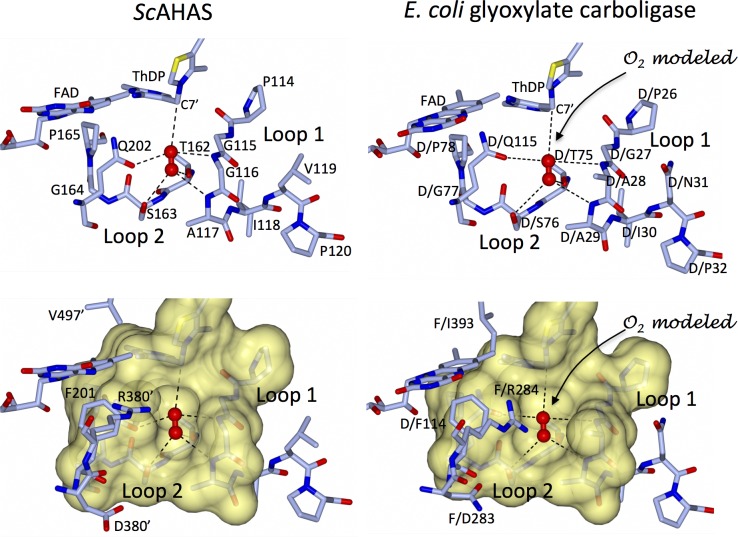
The O_2_(I) binding pocket in *Sc*AHAS and glyoxylate carboligase from *E*. *coli* (EcGCL). The shape, charge and bonding partners are conserved in both enzymes. Dashed black lines represent the interaction network of O_2_(I), including hydrogen bonds and van der Waals interactions.

**Fig 5:**
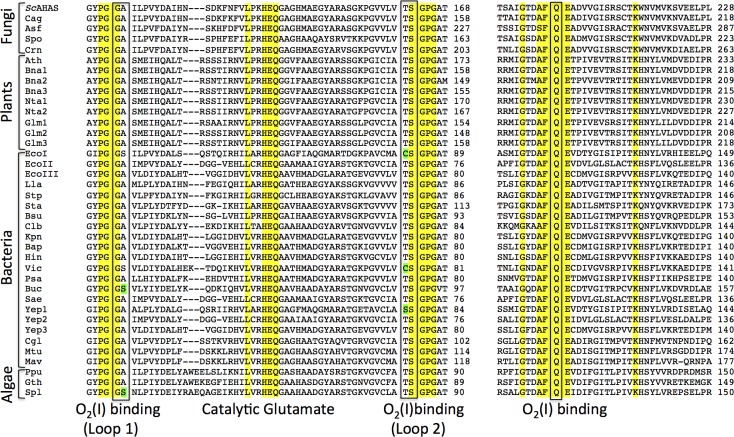
Alignment of AHAS sequences from different organisms highlighting residues in the O_2_(I) binding site. Yellow shading indicates residues that are fully conserved in all the enzymes represented here. Amongst 38 AHAS sequences belonging to all types of organisms producing this enzyme (bacteria, plants, fungi, algae) P114, G115, and G116 from loop_1 and S163, G164, and P165 from loop_2 are fully conserved. Q202, critically important for catalysis, is also fully conserved. A117 and T162 are also highly conserved, being substituted only in three cases. Notably, T162 can be substituted by a cysteine or a serine residue which can play a similar role in interacting with O_2_.

Firstly, O_2_(I) fits perfectly into a well-defined pocket lined by five residues (G116 and A117 from loop 1, T162 and S163 from loop 2, and Q202) and is within van der Waals contact (3.66 Å) of the C7’ carbon atom of the pyrimidine ring of ThDP (Fig 4). All of these residues are highly conserved amongst the AHASs from different organisms (Fig 5), highlighting the importance of this structural feature throughout evolution.

Secondly, loop 1 is rigidified by P114 and P120 and loop 2 by P165 (Fig 4), with the B-factors atoms in loop1 and loop 2 being low compared to those in the rest of the polypeptide (Table 1). The influence of these two residues on the fold of the polypeptide appears to be crucial for maintaining the binding of O_2_(I). The importance of shape complementarity at this site is highlighted by a mutagenesis study showing that the A117V change in *Sc*AHAS leads to substantial loss of enzymatic activity [17]. As suggested by the configuration of the O_2_(I) binding domain (Fig 4), the more bulky valine side-chain would create a steric clash with the hydroxyl group of S163, increasing the distance between loop 1 and loop 2 and thus altering the conformation of the O_2_(I) binding pocket. The consequential loss of activity [17] is testimony to the critical role played by O_2_(I) in the activity of AHAS. Furthermore, Baig et al. [18] have shown that in AHAS from *Mycobacterium tuberculosis* (*Mt*AHAS), the replacement of P126 (analog of P165 in *Sc*AHAS) by threonine, valine, or alanine leads to the loss of 95% of enzymatic activity, again highlighting the need for conservation in this region of the polypeptide for effective catalysis to occur.

Thirdly, a strikingly similar O_2_(I) binding pocket is observed in *E*. *coli* glyoxylate carboligase (*Ec*GCL) (Fig 4), an enzyme that also possesses ThDP and FAD as co-factors. All the structural components forming the O_2_ binding pocket in *Sc*AHAS are conserved in *Ec*GCL. In addition, both enzymes have nearby phenylalanine and arginine side-chains (F114 and R284 in *Ec*GC; F201 and R380 in *Sc*AHAS) that appear to play conserved roles. The preservation of this feature appears to be more than a coincidence taking into account that *Ec*GCL and AHAS are both enzymes for which the presence of FAD is absolutely required, however without AHAS having an assigned role in the catalytic mechanism of the reaction [3]. Thus, it is highly plausible that FAD and O_2_(I) play the same role in both enzymes.

The second molecule of O_2_ found in the active site of *Sc*AHAS, referred to as O_2_(II), is poised over the thiazole ring of ThDP (Fig 2), in a fashion similar to that of the binding of O_2_ to FAD in bluB, an enzyme involved in vitamin B12 biosynthesis [19]. Thus, both O_2_ binding sites have previously been visualized in other enzymes, but here for the first time we also observe the locations of O_2_ binding to AHAS.

The B-factors for O_2_(I) and O_2_(II) are higher than the average value of the polypeptide (Table 1). In the case of O_2_(II), this may be explained by the fact that only few interactions coordinate this oxygen molecule to ThDP and water molecules, and, in CC_1, to M582. The elevated value of the B-factors for O_2_(I) is harder to rationalize as this oxygen molecule is involved in a dense interaction network with five residues from Loop 1 and Loop 2. However, since dioxygen is a non-polar molecule the interaction forces are mainly induced dipole interactions, which are comparatively weak. We believe that the absence of stronger interactions contributes to the observed elevated B-factors.

### O_2_ tunnel

**Fig 6:**
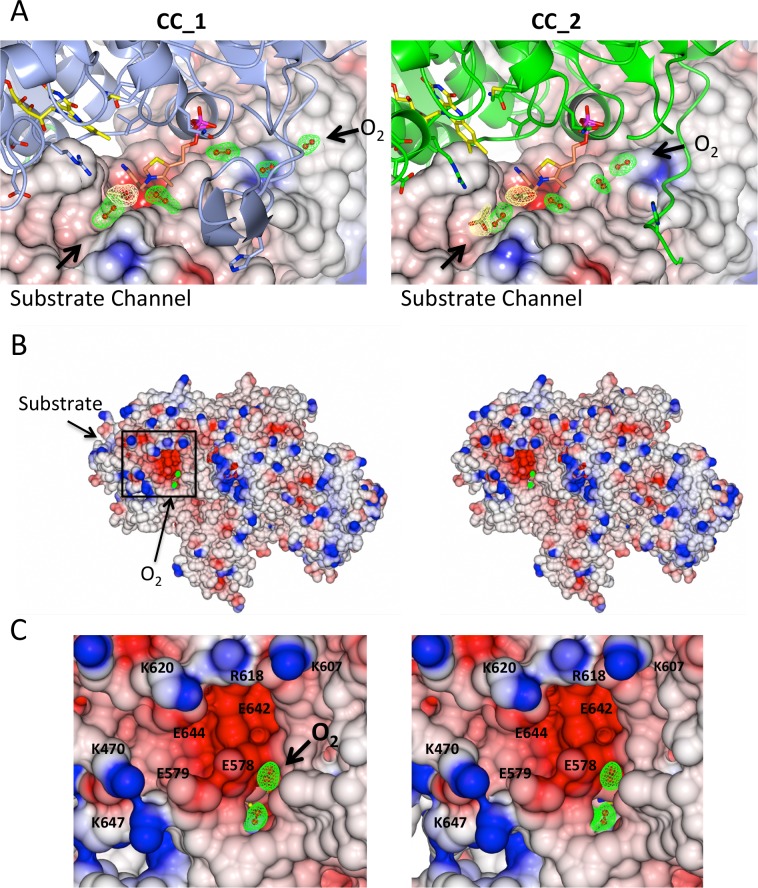
Stereo views of *Sc*AHAS showing that substrate and O_2_ access the active site by different routes. (A) Overviews of CC_1 and CC_2 showing that substrate and O_2_ have different routes to access the active site. (B) Electrostatic potential surface of *Sc*AHAS. (C) inset of (B) focusing on the entry of the O_2_ tunnel that connects the solvent exposed surface to the active site. (A) to (C) Green electron density contoured at 3.5 σ in a F_o_—F_c_ map for the O_2_ molecules. (A) Lemon electron densities (2F_o_−F_c_) correspond to acetate molecules contoured at 1.5 σ (acetate closest to ThDP in CC_1 and CC_2) and 1.2 σ (second acetate in CC_2).

In addition to the presence of O_2_(I) and O_2_(II), additional O_2_ molecules (three in CC_1, two in CC_2) could be fitted to the density though disordered water molecules could, to some extent, also be fitted to the same density (Fig 6A). However, additional elongated densities were also present in the same positions for up to 15 independent data sets that we collected where we soaked pyruvate over a range of times and concentrations into these crystals. These data give further credence to the assignment of O_2_ molecules in the locations closest to the active site. Furthermore, nowhere else in any of the structures could we assign such elongated densities that could be signified as O_2_ molecules instead of water molecules. Importantly, this arrangement shows that O_2_ and pyruvate do have different routes to the active site, with O_2_ progressing along a well-defined tunnel from the solvent exposed surface to the active site (Fig 6). In further support of this idea, a similar pathway for the movement of O_2_ is also observed in the structure of L-amino acid oxidase [20]. An analysis of the electrostatic surface shows that the O_2_ tunnel has a well defined organization with negatively and positively charged amino acids alternating through its length (Fig 6C). Such an arrangement would facilitate transport of O_2_ through the tunnel.

**Fig 7:**
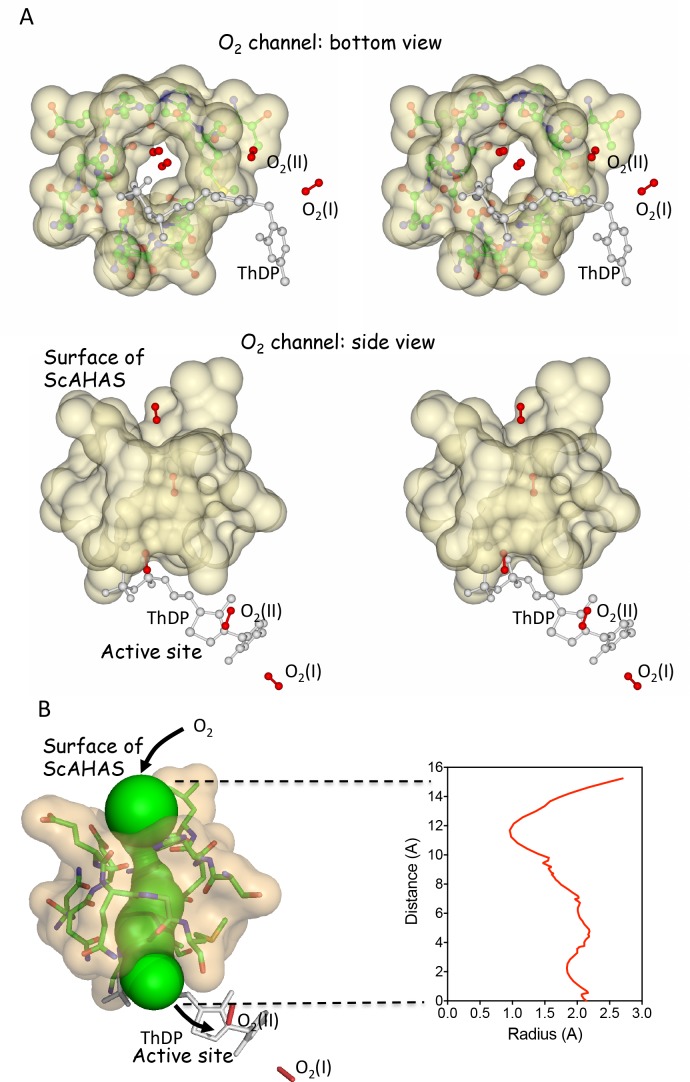
Characterization of the O_2_ tunnel. (A) The *Sc*AHAS structure showing residues in the vicinity of the tunnel in CC_1. (B) Computational analysis of the O_2_ channel showing solvent accessibility (Green color) and corresponding radius.

A representation of the structure shows a well defined channel from the surface to the active site of the enzyme (Fig 7A). Computational analysis using MOLEonline 2.0 (21) shows that this predominantly hydrophilic channel is 15 Å long, and measures 2 Å width at its narrowest point (Fig 7B). The van der Waals radius for oxygen in molecular oxygen is 1.2 Å and taking in account the flexibility in the torsion angles of the long side chains of residues (D550, N600) in this region, it is highly plausible that O_2_ can pass from one end of the tunnel to the other.

The occurrence of a pathway specific for O_2_ is in complete agreement with a previous study based on a competitive enzymatic assay with O_2_ and pyruvate, that shows O_2_ must use a path to access the active site that is different from the one used by the acceptor substrate [5]. Thus, even in the absence of definitive density for the O_2_ molecules there is strong corroborating evidence that such a path for entry of O_2_ to the active site exists in AHAS.

## Conclusions

The new high-resolution crystal data for *Sc*AHAS reveals a number of novel insights into the structural and functional features of this enzyme. These include:

1. The general asymmetry of the dimer reflecting the different conformations taken by FAD in the catalytic centres, possibly related to different redox status.
2. The presence of two O_2_ molecules in the active site, with O_2_(I) proposed to act as a co-factor having a role in AHAS catalysis.
3. A tunnel that allows O_2_ molecules to access the active site independently of substrate avoiding proximity to FAD.

Further studies including kinetics and structural investigations of *Sc*AHAS in the presence of substrates, and electron paramagnetic resonance measurements for the detection of radicals is required in order to investigate the specific roles for O_2_(I), O_2_(II), and FAD in AHAS catalysis.
